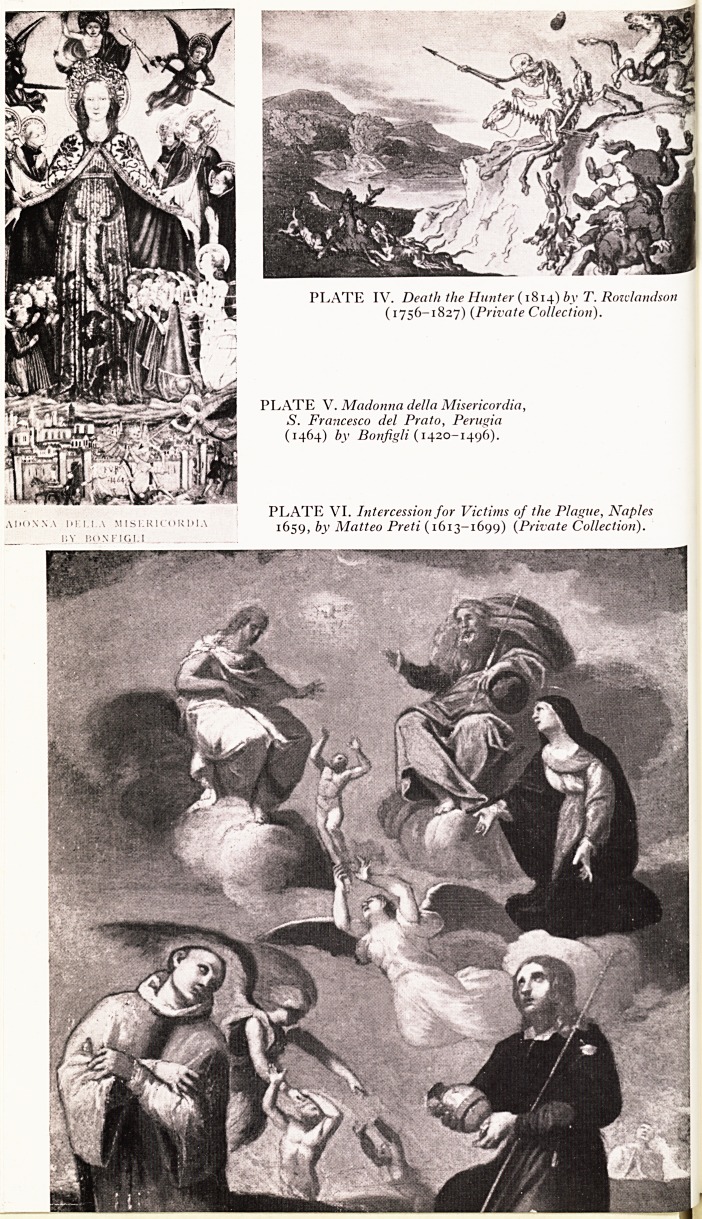# Society, Artists, and the Plague

**Published:** 1966-01

**Authors:** R. E. Hemphill

**Affiliations:** Lecturer in Mental Health, University of Bristol, Consultant Psychiatrist, Glenside-Barrow and United Bristol Hospitals


### SOCIETY, ARTISTS, AND THE PLAGUE,
### *with an account of the Plague in Bristol*

BY

R. E. HEMPHILL, M.A., M.D., D.P.M.

*Lecturer in Mental Health, University of Bristol,*
*Consultant Psychiatrist, Glenside-Barrow and United Bristol Hospitals*

> "Plague added the finishing touches to the great misery of Bristol; its inhabitants resembled prisoners more than citizens, being so low with taxation, so poor in habit, and so dejected in countenance." (Skeete 1645, quoted Latimer)

Three hundred years ago the final and most dramatic epidemic of the plague struck England, and with the terrible visitation at Marseilles in 1720, epidemic plague disappeared from Europe after 400 years of domination since the Black Death of 1348. Between these dates epidemics were so frequent that every generation must have thought them inevitable. That man could have renewed the task of rebuilding society after each is a testimony to the optimism and the indestructibility of the human spirit, and especially in Europe.

The epidemiology and history of plague have been well documented, but less attention has been paid to the psychological than to the social and medical aspects. In commemorating the anniversary of the last visitation, and the three hundred years of freedom, it is relevant to enquire how society reacted in the post-epidemic phases as well as during the attacks. With the sense of security that science and modern medicine give, it is now difficult to imagine the effect of this recurring, irresistible and incomprehensible horror on thought and outlook.

Writers, artists and architects, who express the feelings and aspirations of their generation, have left their testimony. Petrarch, Boccaccio and Machiavelli were eye witnesses of the Black Death, Pepys and others of the 1665 visitation in London, while Defoe's moving and accurate *Journal of the Plague Year* is derived from the epidemic at Marseilles in 1720, not from London as it pretends to be. The experience of Bristol, a city that suffered often and severely, can be found in contemporary records.

Plague, bubonic and pneumonic, existed from ancient times, and was probably the pestilence that destroyed armies, referred to in the Old Testament. There were frequent outbursts in Europe and in England before the fourteenth century, but in scale and severity the Black Death of 1348 was quite different. In two years hardly a town was untouched, over 200,000 were depopulated. Florence lost over 100,000, and England half of its population in a generation. Cyprus and Iceland are said to have been totally depopulated, and the death roll for the civilized world was estimated at 43 millions in two years.

Plague travelled from the East along the main arteries of commerce, and entered Messina in twelve Genoese galleys in October, 1347. The mortality in Sicily was immense. Early in 1348 two ships from Messina brought the Black Death to Pisa on the mainland. It passed through Tuscany, Northern Italy and across the Alps, and in August 1348 hit England. Thereafter there were frequent and severe outbreaks on the Continent, and in England, where the worst years were 1569, 1593, 1603, 1625, 1636 and 1665, scarcely a five-year period up to 1670 was free from desolation.

The plague caused stabs of pain like arrows, gangrenous inflammation of the throat and lungs, foul body smell, purple spots, painful buboes that ulcerated and discharged. The incubation was three to five days, and mortality about 80 per cent. It was impressive for the rapidity with which it struck and killed, the sufferings of the victims, the stench of decay and death, and the extreme contagiousness. The epidemics must have been mixed, and no doubt some of the deaths and recoveries regarded as plague were due to other acute illnesses.

Before 1348 plague was mainly pneumonic, thereafter bubonic or both together, with a double risk of transmission, direct and by fleas. When it had passed a city, few of the inhabitants were left alive and the appearance of the empty towns with houses and contents intact must have been uncanny. London in 1665 was like a desert.

Plague was believed to be God's punishment for wickedness acting through physical agencies, such as vapours, the air, stenches, poison, insects, and the work of the devil who was released by Him for the purpose, as a dog might be released from a chain to attack a trespasser. Even Hippocrates had thought it was of divine origin. Accordingly its causation and prevention seemed to be ecclesiastical matters, the management of the epidemic secular. City Councils quickly legislated for quarantine, segregation of sick and their contacts and suspects, and building pest houses in order to control the epidemic and prevent the spread. Improved sanitation came later. Quarantine was so called from the forty days of Christ's temptation in the wilderness.

The sick and their families were confined to their homes, which were marked with a red or white cross, and later suspects were incarcerated in pest houses that were set up everywhere. Venice formed the first sanitary council, and Padua in 1577 after 2,000 deaths was the first to appoint plague physicians or Medical Officers of Health. Bristol appointed its first Medical Officer of Health three hundred years later, after the cholera epidemics.

Soon all countries evolved elaborate regulations for controlling goods, animals and humans, said to have been more extensive and complicated than for the conduct of the wars, but they could not be fully operated nor enforced for want of officials, nor publicized when there was no official left alive who could write.

To the confinement of suspects and restriction of travel were added the enormous tasks of disposing of the dead, attending the sick, and destroying "sources of infection". The dual office of nurse and gravedigger was usually vested in the same individual, often a released criminal; on the Continent galley slaves, who preferred the risks of freedom to the certainty of living death in the galleys. They were also "searchers" who looked for the marks of plague on suspects. Called Menotti, or Becchini from the iron hooks with which they dragged out bodies, they were everywhere feared. They were accused of killing and robbing the sick and of breaking into the houses of the healthy to extort money under the threat of dragging them off to the pest houses. For a consideration they would roll the dead in damp clothes to cause the plague spots to fade, so that the cause of death could be registered otherwise.

Epidemics over, the Menotti were often punished, whether justly or not cannot be estimated, for few victims or eye witnesses survived to testify.

Bodies were stacked in mass graves and plague pits, or dumped in harbours and rivers. In Avignon, where 120,000 died in 1348, including Petrarch's Laura, Pope Clement consecrated the Rhone for that purposes.

Nursing was negligible, but the segregated did get food somehow. So great was the fear of contamination that the Host at Communion was served on a long wooden spoon, shoppers picked up their goods and dropped the exact sum of money into a pot of vinegar, in England Wills were dropped from the windows to clerks in the street, and in 1665 Pepys wondered if it would ever be safe to wear wigs again as one could not be sure they had not been made from the hair of plague victims.

Plague was in a way a disease of prosperity, as typhus is of poverty, and trade favoured the spread. Wherever men assembled for business or pleasure the risks were enormous from contact and from the rats that accompanied them in ships or on land, attracted by food. The growth of wealth and trade in the fourteenth century is regarded as responsible for the violent spread of the Black Death.

There was great resistance to the regulations to control activity, for they prevented commerce and struck at the heart of collective meetings for social intercourse essential to civilisation and democratic culture. This conflict of fundamental essentials was one of the causes of the continuous social tension responsible for much strange "release" behaviour.

The rich and those who could do so fled from the cities to the country until the epidemic was over. They were welcomed by the tradespeople but not by the others, who feared that they would bring the plague.

Merchants avoided cities where the plague was raging, ships were diverted to other ports, and there must have been considerable competition for trade among the plague-free towns. But business went on somehow and throughout all the great epidemics wars, pilgrimages, meetings and Crusades as well as commerce were not suspended. There was always a temptation to conceal plague, and the epidemic of Naples in 1656 became serious before the authorities would admit it, as this would have closed the port to commerce.

As families perished it was difficult to prove the right to property, and remote connections of a great family might find that they had unexpectedly inherited estates and titles. After epidemics generations of new rich appeared who were less shackled by tradition, and if guilty of vulgarity and extravagance, they also brought a new and vigorous spirit, important in rebuilding society. Although untended herds and fields may have been spoiled, there must have been much accumulated wealth to give the new generation a good start.

### CLINICAL AND PSYCHOLOGICAL

Plague was believed to be a punishment for sin, general and specific, such as the opera (Spain), the theatre (England 1663), long pointed shoes, and fashion. The Black Death was a rider on a Black Horse, and plague was later depicted as the rider on the Pale Horse of the Apocalypse. In some countries a Plague Virgin who took the form of a blue flame on the lips of the dying and accompanied by an owl (a sinister bird of mediaeval thought) scattered infection. The idea that the stabs of pain were caused by invisible arrows is perpetuated in the clinical word toxin.

The disease was present in the air and propagated through the breath or skin. It was a form of poison emanating from the moral evil that destroyed the body from within.

Martin Luther said that pestilence was caused by evil spirits which infected the air and infected people by their breathing. He, confident of his virtue, never avoided the sick or the plague houses, and survived. The air, infected with "tiny creatures" was "denser" than normal. To dispel the infection enormous fires were lit in the streets; bells were rung and cannons and guns discharged to break the "density". These, with the masses of dead and the cries of the Menotti, were familiar sights and sounds of a plague city. Foul smells, dirt, rotting flesh and decay were seen as actual as well as symbolic signs of moral corruption. The greater the stench the greater the guilt.

Comets and other astronomical events were precursors, and since they were bound to occur, epidemics were inevitable. Earthquakes, floods and swarms of locusts added to the horror of some of the epidemics, and monstrous creatures as depicted in the paintings of Bosch were often reported. People were driven mad with terror at the predictions.

Mediaeval theory had to conform with Church teaching, and as the Universities were under the jurisdiction of the Church, physicians were instructed to care for the body and leave the mind and soul alone. Since plague was God's punishment, prevention and causation were not supposed to be the business of the physicians. As might be expected, therapy was mainly ineffective. It aimed at destroying the evil spirits and the aromas and vapours which conveyed them, but no clear distinction was made between evil in the physical and in the moral sense. Indeed they were often believed to be the same.

Among the remedies were incantations, wearing amulets, scent apples, bunches of flowers, annointing the whole house with aromatic oils, sitting between fires, taking snuff and tobacco. In the seventeenth century Eton boys were punished for not smoking. Cheerfulness, music to dispel dreams, and sexual continence were strongly advocated. Paracelsus argued that a cheerful spirit defended the body against disease—"One should not be afraid, imagination sets fears alight".

Tanners and butchers were said to be immune, so contact with hides and tallow and inhaling the breath of a horse were curative. Incidently Dr. Beddoes of Bristol in 1806 treated the consumptives by inhalation of cow's breath in a "breathing tent" which he thought was a folk medicine. Painful buboes were usually left to burst on the theory that the evil spirits would then find their own way out.

A few physicians behaved nobly and with skill, and even died, but in general they had a bad reputation and were often accused of creating rumours of plague to attract patients. In Italy they were apt to be imprisoned for having defrauded the survivors.

The poor were naturally bitter against the rich and the professional classes who had failed them, and even accused them of causing the plague.

### PSYCHOLOGICAL REACTIONS

Great emotional tensions were generated by the exceptional conditions and restrictions, by the disruption of society and the failure of the Church and State, the horrifying environment of the plague-struck towns and by the continuing anxiety and constant contact with death and decomposition. These gave rise to crime and opportunism and also sought relief in abnormal individual and mass behaviour which was motivated by fear, insecurity, aggression, masochism and sadism.

In the Black Death there must have been a reaction first of terror and superstitition then disapppointment and resentment. When it passed there would be a reaction of relief until the next epidemic, then a repetition of the psychological disturbances, and eventually a degree of adaptation and acceptance.

### DEATH

A morbid preoccupation with death almost amounted to a cult, and from the fourteenth century onwards popular art was full of representations of the Dance of Death. Death, which gave relief from the horrors of life, was not entirely to be feared, and a complex death-wish coloured the thinking of the period and was expressed in painting, dancing and literature. The artist seems to side with the enemy in paintings of popular subjects such as "Death the Hunter", "The Dance of Death" or "The Triumph of Death". Death is seen to lead a dance of humans who cannot resist it. Performances of the dance were executed in many cities: they were both formal Dances of Death as well as frenzied mass dancing in the streets. The term "Danse Macabre" originates from a Dance of Death instituted by a Scots adventurer called MacCaber ,"Half a Skeleton" in France in 1424.

### DANCING MANIA

Outbreaks of the so-called dancing mania swept through cities of Western Europe between 1374 and 1614. The dancers would appear to be in a trance, were highly excitable, and danced with contortions. It was said that the colour of red or a pointed shoe would set them off. Exhaustion and death were often the end, and at Strasburg they were sent to the Monastery of St. Vitus. There seems no doubt that many of these dancers suffered from delirium due to an encephalitis, and that their wild movements set off hysterical reactions in others in emotionally charged crowds. In Italy the bite of the tarantula spider was blamed.

Some epidemics of dancing were due to ergotism, the result of eating contaminated flour that had been neglected when millers had died in the plague. The delirium athetosis and involuntary movements stimulating dancing are well depicted, by Peter Breughel (Plates I and II).

This morbid pre-occupation with the idea of death continued far beyond the plague days. The Vanitas Still Life (Plate III) was a popular subject in Netherlands painting to the end of the seventeenth century. In this a skull and objects were arranged so as to underline the inevitability of death and the futility of human aspirations.

Even the lively Rowlandson expressed this interest in his Dance of Death series (Plate IV).

Giorgione (1510) clung to the lips of his lover, sick with the plague, as three hundred years later the romantic Liszt did to Marie du Plessis, "la Dame aux Camelias" herself dying of phthisis. Giorgione and his lover perished.

### MASOCHISM AND FLAGELLATION

A sense of collective guilt coloured popular feeling. It expressed itself in the masochistic urge for self punishment and humiliation. This masochism, which seems to have no sexual element, reached great heights with the Flagellants. They travelled from Dresden to London, attracting thousands. The Flagellants would stop in the public squares of the towns *en route*, and after prayer and ritual would flog themselves savagely, identifying themselves with the sufferings of Christ. The emotional public would do the same, and many joined the movement.

Probably masochism and self-degradation were responsible for some of the revolting therapies. Patients would drink urine and pus, make concoctions of boiled urine and excretions, inhale bottled flatus and sleep in manure heaps. Hossman, quoted by Nohl, advised, "A wash with urine does more than any preventative, most particularly when in addition the urine is drunk". The latter was said to be favoured by grave diggers. Rommell, also quoted by Nohl, recommends drinking morning urine and "inhaling the stink of privy places, and daily bathing in urine to preserve one from the greatest danger".

Patients would plaster buboes and their heads with disembowelled toads and the entrails of other animals to draw out the poison. All these therapies were a blend of homeopathy and self-punishment.

### AGGRESSION AND SADISM

Aggression found its outlet in companies of Luciferian and other anti-God sects who roamed Europe, committing terrible atrocities, even cannibalism, and preaching atheism.

The often reported acts of the sick deliberately infecting the healthy with pus from their buboes were also motivated by aggressive urges.

Similarly citizens, disgusted at the ineffectiveness of the Church and the doctors, broke with morals and advocated pleasure with fatalism. In some cities, as a reaction, girls wandered naked in the street, and it was advocated that women, ill from whatever cause, should be stripped and inspected in every part by a man, an idea quite shocking to the mediaeval and Renaissance code of decency.

Sadism found ready expression in the search for scapegoats, the great witchhunts, and the persecution of the Jews. On the Continent these horrors continued until the eighteenth century. Many innocents were accused of putting "plague powders" on the doors to disseminate infection, acting for the devil. They were invariably found guilty and savagely tortured to death in public. Notable cases occurred at Milan, Naples and Geneva. The Church and secular authorities encouraged these accusations, hoping to shift the blame for their failures on to others. "Plague Smearing" was a common charge in the witch trials.

### SUPPLICATION

But the general instinctive reaction was to appeal again and again to the Church for help. Services and Ceremonies of Intercession were held, and the Church became enormously rich from offerings it received. To a Christian the greatest dread was of dying unconfessed without the last rites, like Hamlet's father, for this might condemn him to an eternity of damnation. The circumstances and suddenness of plague deaths almost invariably prevented these ministrations. Life was short, eternity long, and to die unconfessed and without absolution was the ultimate horror. As soon as the plague had passed, Services of Intercession for the souls of the dead were held.

In the sixteenth century, mystery and miracle plays were common. Their object was partly to appease God, and later they became a regular form of intercession.

### ARTISTS AND THE PLAGUE

There were many patron Saints of plague victims, the most celebrated were S. Sebastian (date unknown), identified in painting by the arrows with which he was killed, and St. Roch (fourteenth century). St. Roch of Montpelier actually tended the sick and died of plague. He can be recognized by the incision of a buboe on the left thigh. These Saints were depicted in "plague pictures", many of which still exist, by Titian, Corregio, Guido Reni, Raphael, Preti, Gozzoli, Sodoma, and other important artists.

Plague pictures were not intended as records, but were partly votive, partly intercessional. They were carried in processions and deposited in the Churches as offerings. They have a special place in art. Inspired by the feelings of tragedy the artist experienced, they express the special sufferings of the plague of the invocation of the Madonna and Saints. Perugia was famous for its Gonfaloni, or expiatory and votive banners. Offering representations of affected organs or objects of disease to the healing God is ancient magic—some made to Asklepios can still be seen at Epidauros. Golden mice and golden emerods (buboes) were offered to the Ark after a plague devastation of the Philistines (1 Sam. VI). In the plague paintings representations of the emotional sufferings, as well as the physical disease, are offered as expiation with an appeal to the Saints depicted to intercede with the Almighty.

In the example by Bonfigli (1464) (Plate V), arrows of plague break ineffectively on an oversize Madonna. Above her Christ casts arrows down, on his right is the Angel of Justice with a drawn sword. on his left the angel of Mercy sheathes a sword. Below is S. Sebastian pierced by arrows. Death draws a bow outside Perugia as the Archangel strikes him with a spear.

The very moving picture (Plate VI) by Preti (1559) shows on the left St. Vincent in the robes of a Deacon, and on the right St. Roch with the shell and staff of a pilgrim, the incision of a buboe on the left thigh, the palm of penitence on the ground. Angels are raising the souls of the dead from the plague pits of Naples and passing them up to the Madonna and Christ to intercede for them with God the Father, and the Holy Spirit in the form of a Dove.

Intercession for plague is still included in the Book of Common Prayer of the Church of England, and links plague with the idea of sin and divine wrath. "From plague, pestilence and famine . . . from Thy wrath and from eternal damnation, good Lord deliver us".

Perhaps because they were unable to leave their workshops in the towns, many artists died of plague. Among the more celebrated were the two Lorenzetti (1348), Ghirlandaio (1494), Giorgione (1511), Perugino (1523), Holbein (1543) and Titian (1576).

### AFTER THE BLACK DEATH

"Oh, happy posterity, who will not experience such abysmal woe and will look on our testimony as a fable." So wrote Petrarch to a friend after the devastation of Florence in 1350. Little did he know how soon the experience was to be repeated.

Boccachio's Decameron was introduced with an account of Florence—"In 1348 there made its appearance that deadly pestilence, whether disseminated by the celestial bodies, or sent on us mortals by God in His wrath by way of retribution for our iniquities . . . How many grand palaces . . . were now left desolate. How many historic families found no scion to continue the succession. How many brave men, fair ladies, gallant youths, broke fast with their kinsfolk, and when evening came, supped with their forefathers in the other world!"

The Black Death and every subsequent visitation caused a great loss of life amongst craftsmen, artisans, educated, executive and professional classes and priests, as well as the poor. Lay confessors were appointed because of the mortality of the ordained.

One wonders how many were left of a mainly illiterate population who could write, how records were kept and business renewed. Yet within the next century the greatest buildings in Florence were constructed, and in three centuries of plagues all the great works of art in painting, sculpture and architecture of the Renaissance and Baroque periods were achieved. It was as if death rapidly produced new life and ideas. The great riches donated resulted in new and magnificent churches. The question still remains, where did the unskilled labour and the skilled and educated manpower come from?

Trade Unions and co-operative groups were formed after the Black Death, and included even lay brothers of Monasteries. For the next century they took great advantage of the labour shortage. Mullett cites certain benefits which may have resulted from the plagues, public health action was stimulated and there was eventually a higher standard of medical education and practice, the Black Death "shattered decaying institutions and strengthened new tendencies". Still the devastation and cruelty that went with it was a hard price to pay.

The statistics of mortality have been calculated from the burial registers in the Parish records and similar sources, provided there were survivors who could write. The figures are approximate, but at the best indicate about a 30–50 per cent reduction in the population of cities after each attack. Against this is to be set the normal high morbidity and short expectation of life by modern standards, and the dispersal of persons from plague cities who never returned. These figures would be included in the total for any epidemic. There is also no doubt that some villages, even in England, totally disappeared.

After the Black Death there was a flood of marriages, and, it is said, a high fertilit with an unusual number of multiple births and good infant survival. Hope was expressed in fashion as well as in literature, song and music. Men wore tight-fittin clothes, and a short coat to show the legs was introduced for the first time. Colour were bright, and sometimes different for the two legs and sides of the body. Styles o shoes and jewels were extravagant. Women developed a plunging neckline to emphasize their figures. All this gaiety was criticized as evidence of sin and led to gloom forecasts of retribution, that appeared soon to come true.

Yet the optimism and desire for entertainment were irrepressible. Theatre obviously danger spots, were closed in London in 1574, but had to be reopened, an in 1604 performances were permitted provided the audience was not more than thirt quite a big number for the day.

Throughout these three centuries the balance of feeling swung between morbidit and despair, and rebirth and gaiety; and the spirit of man, his resolution and potential were reflected in the art of the Renaissance.

### BRISTOL

The course of the plague in England corresponded with that on the Continen but the extremes of hysterical and emotional behaviour did not occur, and the interes was more on commercial life. Perhaps for this reason the regulations were neither s stringent nor so successful in England until the mid-seventeenth century.

The geographical and commercial situation of Bristol exposed it particularly t plague. The second port, and one of the largest cities in England, a centre of commerc with great annual fairs, it attracted visitors and trade. The confluence of rivers wit marshes and hills on which it was built, the warehouses and conduits, were favourab to vermin and infection.

Fresh water was supplied from the "Boiling Well" at Ashley in a long unprotect conduit to the Quay, and this conduit was frequently obstructed by the bodies of dea cats; there is a receipt for December 1575 "Paid for taking three cats out of the Ke pipe where one was two yards long. Five days 5/6d." If the cat's length seems excessive the time taken to remove it is even more so, a characteristic of the healt service in the sixteenth century as in the twentieth. One wonders how the account wa passed for payment.

The following account of the plague in Bristol is compiled from Ricart's Kalenda Latimer's Annals of Bristol, information supplied from the City Archives, and othe sources.

Not much is known of the Black Death and the epidemics of the fifteenth centur but there were many outbreaks in the sixteenth and seventeenth centuries, and th trade of the city was seriously affected. This is possibly why the precautions and regulations laid down by the City Council did not have much success, although Bristo established segregation and quarantine early in each epidemic. The picture of li and civic management was as elsewhere, except that Bristol was particularly vulnerab as merchants transferred their trade from the capital, and refugees escaped westwar with every major outbreak in London.

Observations for the years 1575–1605 are quoted from Ricart's Kalendar.

1575 "This yeare began the plague to be very hotte about St. James tyde, and betwee St. James tyde and Powles tyde there died aboute 2,000 persons." (That i between July 25th and January 25th. Population of Bristol ?15,000.)

1603 "This yeare in the moneth of July the greate plague began in this Citie, an contynewed eighteene monethes, and from July untill the xxixth of Septembe followinge there died of the plague one hundred persons or thereabouts."

PLATES I AND II. *The Dancing Mania after Pieter Breughel, engraved by H. Hondius* 1642 (*Collection of Author*).

PLATE III. *Vanitas Still Life with child blowing bubbles by Jakob de Wit* (1695–1743). (*Private Collection*)

PLATE IV. *Death the Hunter* (1814) *by T. Rowlandson* (1756–1827) (*Private Collection*).

PLATE V. *Madonna della Misericordia, S. Francesco del Prato, Perugia* (1464) *by Bonfigli* (1420–1496).

PLATE VI. *Intercession for Victims of the Plague, Naples* 1659, *by Matteo Preti* (1613–1699) (*Private Collection*).

(1603–04 was an appalling plague year. London lost 34,000 out of a population of 225,000, Bristol 2,240 and Bath 70.)

1604 "This yeare there dyed of the plague in this Citie 2,950 persons."

1605 "This yeare there dyed of the plague in this Citie 150 persons."

1610 An outbreak occurred in the autumn and continued until the following summer. The mortality on this occasion is not recorded.

1611 Another outbreak, when a large number died. The total figure is not known, but 81 victims died in the week ending September 23rd. Another pest house was established.

1625 It was said that infected persons with open sores had packed cloth and sent it to Bristol, thereby infecting the Army in Ireland, for which Bristol was the port.

In June the Corporation forbad Londoners from attending the Great Summer Fair because of the plague in London, and all goods from the capital had to be "aired" for a month outside Lawford's Gate before admission, and the same applied to any citizen returning from London before he would be readmitted. In consequence the city escaped desolation. Watchmen were on guard night and day at a cost to the city of £250.

1636 The Bristol fairs, particularly St. James' Fair, were of great importance. Drapers, skinners, upholsterers and other tradesmen attended it from all over England, Ireland and Wales. As the plague was again in London an attempt was made to shut the fair, but since this would have lost the city enormous commerce, traders were permitted to enter on production of certificates of health from the Lord Mayor. There is a suggestion that there was a black market in the certificates. Similar regulations were enforced in January and July, 1637.

1641 There was another scare due to the outbreak of plague at Taunton, and it is recorded that a physician and barber received £20 for looking after suspected cases. They both fell ill, survived, and subsequently the doctor was paid £4 and the barber £10 as compensation for the loss of their business.

1645 There are no accurate figures for the mortality, but in 1645–46 they are said to have been "fabulous".

In 1645 so many died in one week in the parish of St. Nicholas that it was not possible to record their names, the clerk and sexton having also died, despite the fact that sixteen years earlier the parish had contributed the highest amount in rates towards the cost of cleaning the streets. (E. Ralph.)

Latimer writes "The horrors of pestilence were now to be added to those of Civil War—the plague appeared the previous autumn and the Corporation hired Knowle House to which to send infected persons—the sickness was not then serious. Next April a pest house of thirty huts was established for suspects who had to remain thirty days. Large numbers of poor were there segregated in great want and anxiety; loans were taken up for their assistance, and £100 advanced by Alderman Farmer remained owing for thirteen years because of the city's penury".

Cromwell set up headquarters in Bristol although the plague was raging. He stated in a letter that his army, although quartered in infected places, lost only one man. Whether this is a tribute to their hygiene or their faith is debatable.

In the spring of 1645 William Kemp, M.A., of Bristol published a "Brief Treatise of the Nature of the Pestilence", and one of the King's Chaplains in Bristol in a sermon warned women that the fashionable black patches called "beauty spots" were forerunners of plague spots. When the plague soon broke out the "patched" women were driven out of the city.

1650–52 The plague was serious. The Council leased Old Park, St. Michael's Hill, where they built huts as pest houses. In consequence it acquired the name [illegible] Stinkards Close, modified later as Tankards Close.

From 1649 to 1664 London was almost plague-free.

"In November 1652 an edict was made by the Council that all goods comi[illegible] to the city must be 'aired' for a time at Hollow Backs, near the mouth of t[illegible] Avon, before being brought into the city. 'Hollow Backs' is shown on the m[illegible] of the River Severn between the mouth of the river Avon and Aust." (E. Ralp[illegible]

1665 In June 1665 the Great Plague was raging in London. With the approach [illegible] St. James' Fair the Corporation issued Orders prohibiting holding it and S[illegible] Paul's Fair, and passed various resolutions to keep out the disease. All hous[illegible] holders, armed with halberts, and enrolled like fire watchers and Home Guar[illegible] of 1940, took their turns to keep watch at the entrances to the City, and t[illegible] usual preventive measures were strongly enforced. Nineteen Burgesses we[illegible] on guard duty at a time. Each Burgess took one turn of duty per five week[illegible] from which it has been calculated that there were 1,400 able-bodied citizens [illegible] Bristol. Nevertheless plague broke out in Bedminster and outside Lawford[illegible] Gate, and there were isolated cases in St. Philip's Marsh, where the infecte[illegible] families were shut up in their houses or removed to pest houses.

A pest house was built at Baptist Mills on some land known as "Forlo[illegible] Hope". The mortality has not been recorded, but it must have been insignifi[illegible] cant by comparison with London, for when a fund was set up for the relief [illegible] poor families in London, Bristol contributed £205, Exeter £222 and Taunto[illegible] £155, out of a total of £1,258 raised by the provincial towns.

In the summer of 1665 there were 100,000 plague deaths in London, an[illegible] Pepys recorded that he first saw a "marked" house, and was so disturbed th[illegible] he made a will, "seeing that no man could count on being alive more than tw[illegible] days".

### AFTERMATHS

Isolated cases of plague in Bristol occurred until the summer of 1666. Althoug[illegible] the restriction on commerce as well as the mortality was a serious blow to Bristo[illegible] already ruined by the wars, there were, as always, some who tried to profit.

In October 1666 complaints were made that Bristol merchants were making vas[illegible] profits on their imports due to the destruction of the London stocks by the Grea[illegible] Fire. On June 27th Bath Council asked for a removal of the St. James' Fair from Bristol to Bath, claiming that plague was then prevalent in Bristol, when in fact ther[illegible] were no cases at all outside the patients in the pest houses, an example of oppor[illegible] tunism and sharp practice that was unsuccessful.

A Bristol letter of October 1666 (Latimer) states that thirty ships returning to Vir[illegible] ginia and Barbados would carry "slender cargoes as Bristol goods were bought s[illegible] cheap and sold so dear that a small quantity brought a large return".

With the Great Fire of London in 1666 the history of epidemic plague in Englan[illegible] was practically brought to a close, although one case of plague in Bristol was reporte[illegible] in 1916 during the Great War.

The reasons for the ultimate disappearance of the plague are not certain, but Mulle[illegible] mentions greater personal cleanliness, the use of cotton instead of wool, the disuse [illegible] straw for bedding, the use of brick instead of wood for building, and the arrival of th[illegible] brown rat from Hanover in the seventeenth century, driving the black rat back to th[illegible] ships. Nevertheless drainage and the disposal of sewage and filth continued to be ap[illegible] palling, and Local Authorities were reluctant to admit it and go to the great expense [illegible] tackling the sewage problems. However, minute books in Bristol record that orders wer[illegible]

made for the removal of vast heaps of filth and refuse in eight parishes in Bristol in 1666.

Attempts were made to control domestic animals and their mess, more because of their suspected connection with the plague than for general hygiene. Dogs and cats had been slaughtered earlier. In London, 1572, 656 dogs were killed in one Parish at a cost of 1d. per dog. "Dog and Swine Wardens" were appointed in London to keep ducks from waddling in the pools. No assault seems to have been made on the rat.

In every country people were terrified of the pest houses, and this was the cause of much bribery and corruption. It has been argued they did more harm than good, and no doubt many healthy persons who might have survived were incarcerated as sick or suspects; there to catch the disease and die.

But they were the forerunners of the excellent isolation hospitals of today, essential for the control and scientific treatment of epidemic disease.

Plague imagery is still in the vocabulary of English and European countries. Pest and plague are words now used out of their original context. The conception of a township politically isolated and quarantined was used very effectively by A. Camus in his allegorical novel *La Peste*, about a North African town (? Oran) during the German occupation of France.

For over three hundred years men accepted plague as inevitable, and endeavoured to preserve and develop their way of living in the face of a threat of destruction that they lacked the knowledge to prevent. Today they do likewise in the face of the vaster peril of atomic wars and radiation disease which is of their own contriving and within their own power to eliminate.

It is a sobering thought that but for the one erroneous belief that plague was a punishment for sin, and could be controlled but not prevented, its real origin would have been suspected and its horrors and destruction could have been avoided.

I am much indebted to Miss E. Ralph, Bristol City Archivist, for her comments and help about the Plague records of Bristol.

### BIBLIOGRAPHY

Boccaccio, G. (1368?). *The Decameron*, Vol. 1. Translated by Rigg, J. M. Publ. *Everyman* J. M. Dent, 1963.

Crawfurd, R. (1914). *Plague and Pestilence in Literature and Art.*

Creighton, C. (1891). *A History of Epidemics in Britain*, Vol. I.

Creighton, C. (1894). *A History of Epidemics in Britain*, Vol. II.

Defoe, D. (1772). *Journal of the Plague Year*. Publ. *Everyman*, J. M. Dent, 1962.

Latimer, T. (1908). *Sixteenth Century Bristol.*

Latimer, T. (1900). *The Annals of Bristol in the Seventeenth Century.*

Mullett, C. (1956). *The Bubonic Plague in England.*

Nohl, T. (1926). *The Black Death, a Chronicle of the Plague* (translated from the German by C. N. Clarke).

Ralph, Miss E. (1965). Personal communications.

## Figures and Tables

**PLATES I AND II. f1:**
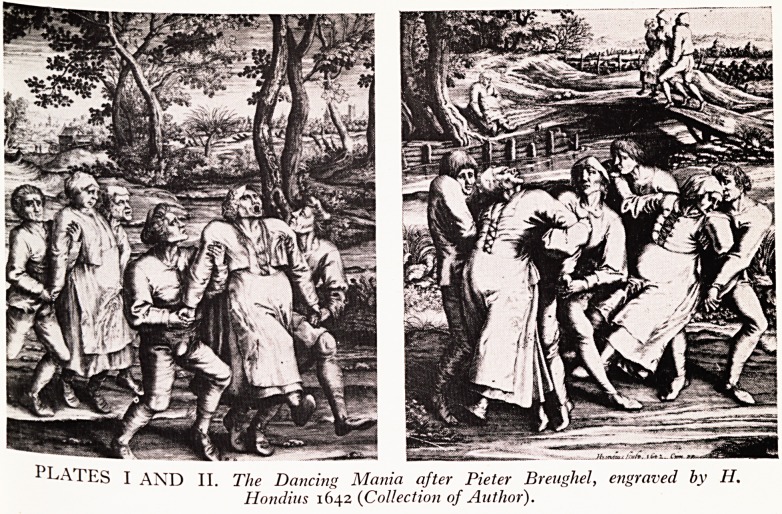


**PLATE III. f2:**
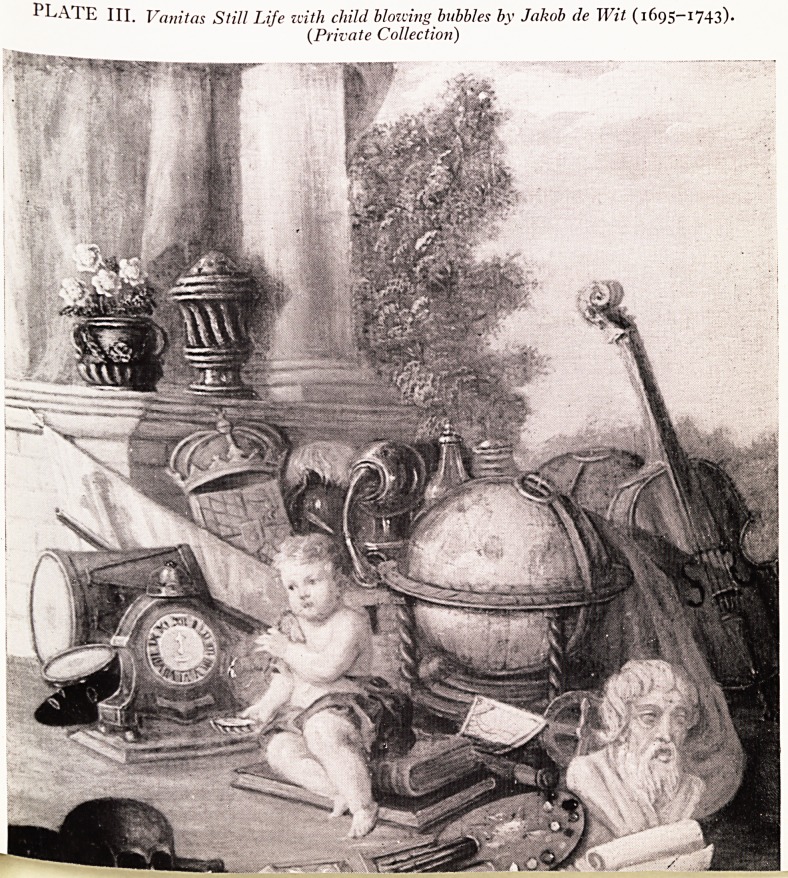


**PLATE IV. PLATE V. PLATE VI. f3:**